# Environmental health risks and benefits of the use of mosquito coils as malaria prevention and control strategy

**DOI:** 10.1186/s12936-018-2412-4

**Published:** 2018-07-16

**Authors:** Jonathan N. Hogarh, Thomas P. Agyekum, Crentsil Kofi Bempah, Emmanuel D. J. Owusu-Ansah, Silas W. Avicor, Gordon A. Awandare, Julius N. Fobil, Kwasi Obiri-Danso

**Affiliations:** 1 Department of Environmental Science, College of Science, Kwame Nkrumah University of Science and Technology, Kumasi, Ghana; 2 Nuclear Chemistry and Environmental Research Centre, National Nuclear Research Institute, Ghana Atomic Energy Commission, P.O. Box LG 80, Legon, Accra, Ghana; 3 Department of Mathematics, College of Science, Kwame Nkrumah University of Science and Technology, Kumasi, Ghana; 4 Entomology Division, Cocoa Research Institute of Ghana, New Tafo-Akim, Ghana; 5 Department of Biochemistry, Cell and Molecular Biology, University of Ghana, Legon, Ghana; 6 Department of Biological, Environmental and Occupational Health, School of Public Health, University of Ghana, Legon, Ghana

**Keywords:** Malaria, Mosquito coils, Indoor air pollution, Risk characterization

## Abstract

**Background:**

Malaria is an infectious disease that causes many deaths in sub-Saharan Africa. In resource-poor malaria endemic communities, mosquito coils are commonly applied in households to repel the vector mosquito that transmits malaria parasites. In applying these coils, users have mainly been interested in the environmental health benefits potentially derived from repelling the mosquito, while oblivious of the environmental health risks that may be associated with exposure to emissions from the use of mosquito coil. This study evaluated the effectiveness of the mosquito coil, ascertained and/or estimated the toxic emissions that may emanate from the coil, and determined its overall appropriateness by conducting a risk–benefit analysis of the use of this strategy in malaria prevention at household levels.

**Methods:**

The repellent ability of mosquito coils was tested by conducting a mosquito knockdown/mortality test in experimental chambers synonymous of local room spaces and conditions. The gaseous and particulate emissions from the mosquito coil were also analysed. Additional scenarios were generated with the Monte Carlo technique and a risk–benefit analysis was conducted applying @Risk software.

**Results:**

Mosquito mortality arising from the application of various mosquito coils averagely ranged between 24 and 64%, which might not provide adequate repellency effect. Emissions from the mosquito coil were also found to contain CO, VOCs, SO_2_, NO_2_, PM_2.5_ and PM_10_. The Hazard Index of the respective pollutants characterized over a lifetime exposure scenario was low (< 1 for each pollutant), which suggests that the concentrations of the specific chemicals and particulate matter emitted from the mosquito coil may not constitute adverse environmental health risk.

**Conclusion:**

Although the risk of morbidity from the use of the mosquito coil was low, the coil yielded limited protection as a mosquito avoidance method. It may, therefore, have a reduced benefit in controlling malaria and should be applied sparingly in a highly regulated manner only when traditionally proven effective vector control strategies are not available or too expensive for resource-poor malaria endemic regions.

## Background

Malaria is caused by an infection with *Plasmodium* spp. transmitted through the bite of the female *Anopheles* mosquito. It is a leading cause of death in many tropical countries, especially in sub-Saharan Africa. In 2016, malaria accounted for 445,000 deaths globally with about 90% of these deaths occurring in the African region [1]. Treatment of malaria usually involves the use of drugs that target the *Plasmodium* parasite in infected individuals, while preventive methods largely involve control of the vector, i.e. the *Anopheles* mosquito. The World Health Organization (WHO) recommended malaria control methods include the use of insecticide-treated nets (ITN) and indoor residual spraying (IRS).

However, the use of mosquito coil as a controlling measure against mosquitoes at the household level is widespread in malaria endemic countries across Africa, Asia and South America. In Ghana, approximately 40% of the population was estimated to apply mosquito coil, with 43% of the users applying them on a regular basis [2, 3]. The mosquito coil is a mosquito repelling incense-like product formulated from a paste of granulated insecticide and a filler, such as sawdust and other solid materials, and then extruded into a coiled shape [4]. Most mosquito coils are formulated to allow approximately 8 h of continuous smoldering, such that the insecticide vaporizes slowly to offer protection against the mosquito. The smoldering effect is associated with gradual release of smoke and emissions that may be undesirable in the indoor environment. Some of the toxic pollutants that may emanate from the burning of mosquito coils and similar incense-like products are CO, VOCs, SO_2_, NO_2_ and particulate matter [5–8].

The continuous application of mosquito coils has, therefore, raised serious environmental health concerns. For instance, exposure to the smoke produced from burning of mosquito coils has been implicated in lung cancer [9, 10]. Shu-Chen et al. found out that almost 50% of lung cancer deaths in Taiwan were not associated to cigarette smoking, but suspected that environmental exposure to the smoke from mosquito coil burning may play a role in the development of the disease. Taiwanese households normally burn coils at home to repel mosquitoes and the risk of getting lung cancer was considerably higher in regular burners of mosquito coils (thrice per week) as compared to those who did not burn any mosquito coil. In Ghana, the use of mosquito coils was associated with self-reported incidence of acute respiratory infection (ARI) [3]. The burning of mosquito coil reportedly produced high levels of indoor PM_2.5_ and CO, which were identified as potential causative factors of adverse respiratory health effects [11].

Thus, even though the mosquito coil is supposed to deliver benefits as a mosquito repellent, it may as well be associated with some environmental health issues, which calls for a systematic risk–benefit analysis to generate evidence to guide future application of mosquito coils as malaria prevention and control strategy. The risk associated with the mosquito coil was assumed in response to emissions from lit coils, while the benefit was in response to the efficiency with which the lit coil repelled mosquitoes. The benefits in terms of mosquito repellency potential could be assessed based on mosquito susceptibility test [12]. On the other hand, the risk of exposure to toxic emissions from lit mosquito coils may be assessed based on a probabilistic risk assessment model [13–15]. If application of mosquito coils is a risky venture, then, people must be appropriately informed and the practice discouraged. However, if the benefits are overwhelming and the risks are within acceptable threshold, then, the mosquito coil could complement the existing options for malaria control, especially in poor communities. This study sought to establish these facts by: (i) investigating the emissions from mosquito coils used locally in Ghana; (ii) assessing the susceptibility of mosquitoes to the lit mosquito coils; and (iii) conducting a risk–benefit analysis to inform policy on the inclusion or otherwise of mosquito coils in malaria prevention and control programmes in developing countries like Ghana.

## Methods

### Mosquito coils

Five different brands of synthetic mosquito coils were used in this study. The mosquito coils were purchased from retail outlets at Adum, a key commercial center in Kumasi, the capital of Ashanti Region of Ghana. The selection of mosquito coils was based on the level of popularity of the product on the local market and the extent of use. General information about the different brands of coils is summarized in Table 1.

**Table 1:** General information of tested mosquito coils

ID No.	Country of origin	Shape	Color	Mass per coil (g)	Active ingredient indicated on package	Burning time (h)
MC 1	China	Spiral	Black	16.5	0.25% esbiothrin	7.50
MC 2	China	Spiral	Black	20.0	0.03% dimefluthrin	9.67
MC 3	China	Spiral	Black	17.5	0.03% dimefluthrin	8.00
MC 4	Spain	Spiral	Black	16.6	0.20% allethrin	8.00
MC 5	China	Spiral	Black	17.0	0.08% meperfluthrin	8.00

### Sampling and rearing of mosquitoes

*Anopheles* mosquito larvae were sampled from stagnant water in communities proximate to the campus of the Kwame Nkrumah University of Science and Technology (KNUST), Kumasi. The larvae were brought to the insectary of Kumasi Centre for Collaborative Research (KCCR) at the KNUST. At the insectary, the larvae were transferred into larval bowls and were further sorted to remove any other species apart from the *Anopheles*. The larvae were reared in their natural habitats’ water since any abrupt change in their environment would be unfavorable and could cause larval mortality. The larvae were maintained in an insectary at 25 °C and 75–80% relative humidity with a photoperiod of 12:12 h and fed with Elite fish flakes meal, which was ground and sprinkled evenly on the surface of their habitats’ water daily. Extra caution was taken to ensure that the fish meal did not form foam over the surface of the water since this could cause oxygen deficiency and lead to larval death. The larval bowls were covered with nets so as to prevent larvae that may emerge into pupa and then to adulthood from flying away. The mosquito larvae went through the four stages, 1st, 2nd, 3rd and 4th instars. After the 4th instar stage, they pupated. Pupae were then collected with a Pasteur pipette into beakers containing water and placed in cages to allow them to emerge as adults. The adult mosquitoes were reared in the cages. All adult mosquitoes were fed on 10% sugar solution imbibed in cotton wool. The cotton wool was changed every 2 days to prevent fermentation of the sugar meal. Three-to 6-day old non-blood fed female adult mosquitoes were then collected and their susceptibility tested against the mosquito coil. For the purposes of the susceptibility test, adult female *Anopheles* mosquitoes were separated from the males. The proboscis of the female mosquito is comparatively smoother and not bushy, whereas the male mosquitoes have a feather-like proboscis.

### Experimental chambers

The mosquito mortality test and the gaseous emissions test were undertaken in experimental chambers synonymous of local room spaces and conditions. The experimental chambers were constructed with wood. There were three different room sizes with dimensions 2 × 2 m, 3 × 3 m, and 4 × 4 m, and a uniform room height of 2.12 m. Each room was fixed with a door of dimensions 197 × 76 cm and two windows, each of dimensions 87 × 65 cm. The doors and windows were protected with net. The floor of each room was laid with nylon carpet, over-laid with white papers. Experiments were conducted under ventilated conditions (with windows opened) and poor ventilation (with windows closed). The average temperature and relative humidity in the experimental rooms are indicated in Table 2.

**Table 2:** Average temperature and relative humidity in the experimental rooms

Room size (m^3^)	Ventilated condition		Poorly ventilated condition	
	Temperature (°C)	Relative humidity (%)	Temperature (°C)	Relative humidity (%)
8.5	28.37 ± 1.96	70.77 ± 5.07	30.08 ± 1.90	66.24 ± 3.15
19	28.56 ± 0.81	71.77 ± 4.30	30.62 ± 1.40	66.10 ± 4.33
34	28.65 ± 0.81	69.53 ± 3.96	27.90 ± 0.54	67.09 ± 4.29

### Mosquito mortality test

Prior to commencement of the experiment, 50 female *Anopheles* mosquitoes (3–6 days old, sucrose-fed) were released into each of the rooms without the lit mosquito coil to serve as a control. The control mosquitoes were treated in the same way as the exposed mosquitoes; they were tested under the same conditions. The objective of the inclusion of the controls was to provide an estimate of natural mortality during the test and also to account for all variables that may induce mortality other than the mosquito coils being tested. Afterwards, one brand of the coil, placed on a metal stand provided inside the coil packet, was lit and placed at the center of each room and 50 female *Anopheles* mosquitoes (3–6 days old, sucrose-fed) were gently transferred from their cages into the experimental room. The experiment was carried out at night (from 6 pm until dawn of the next day) and was repeated in triplicates for each brand of mosquito coil. Considering all the experimental set-ups, a total of 900 mosquitoes were exposed to each brand of mosquito coil. Four days was allowed in-between tests to allow for complete removal of insecticide residues from previous test that could influence new test. At the end of the burning period of the mosquito coils, the number of knock-down mosquitoes fallen unto the white floor of each room were counted and recorded. An adult mosquito was considered to be alive if it was able to fly, irrespective of the number of legs left. Mosquitoes that had lost their wings and could no longer fly were considered moribund and were therefore counted as dead. The corrected percentage mortality was calculated by using Abbott’s formula [16].

$${\text{Corrected mortality (\%)}}\, = \,\frac{{{\text{Mortality}}\,{\text{in}}\,{\text{treatment}}\;(\% )\, - \,{\text{Mortality}}\,{\text{in control}}\;(\% )}}{{100\, - \,{\text{Mortality}}\,{\text{in}}\,{\text{control}}\,(\%)}}\, \times \,100$$

### Emissions from mosquito coils

#### Gaseous emissions

Gaseous pollutant emissions were measured using Aeroqual Series 500 (S500) gas monitors (Aeroqual Limited, Auckland, New Zealand). First, the monitors with the appropriate sensor heads were placed in the experimental rooms without lit mosquito coils to know the level of gases in the rooms prior to burning of mosquito coils in the rooms. This served as control and helped to determine the actual amount of emissions attributable to the coils. The gases monitored, sensor type, sensor range and the minimum detection limits are indicated in Table 3. The CO, NO_2_ and SO_2_ sensors are electrochemical gas sensors (Table 3). The working mechanisms of the electrochemical ambient gas sensors are electrochemical reactions within the sensors. The reaction between the sensor and the ambient gas molecules produces an electrical signal (current) proportional to the concentration of the ambient gas. The volatile organic compound sensor, on the other hand, is a photoionization detector (PID) (Table 3). The PID uses krypton filled UV lamp to ionize TVOC gas molecules and generate a current that is proportional to the TVOC concentration. The gas sensor heads were calibrated using Aeroqual R42 calibration kit, designed and supplied by Aeroqual Limited, 460 Rosebank Road, Avondale, Auckland 1026, New Zealand. The relative errors are 10% of reading. Gas emissions from the mosquito coils were monitored continuously in the indoor environment until the coil has completely burnt out. The measurements were logged at 5 min intervals and average concentrations were estimated for approximately 8 h of monitoring. Logged data were downloaded onto a computer using Aeroqual S500 Gas Monitor Software version S500 V6.4.

**Table 3:** Specifications for Aeroqual ambient gas sensors

Gas	Sensor type	Sensor range (mg/m^3^)	Minimum detection limit (mg/m^3^)
CO	GSE	0–123	0.25
TVOC	PID	0–70	0.03
NO_2_	GSE	0–2	0.01
SO_2_	GSE	0–28	0.11

*GSE* gas sensitive electrochemical, *PID* photo ionization detector

#### Particulate matter

Indoor PM_2.5_ and PM_10_ levels from the burning of mosquito coils were measured using HAZ-DUST Model EPAM-7500 Monitor (Environmental Devices Corporation, USA). The device uses the principle of near-forward light scattering of an infrared radiation to immediately and continuously measure the concentration of airborne dust particles. The monitor with the appropriate impactors was placed in the experimental rooms without lit mosquito coils to know the level of particles (2.5 and 10 µm) in the rooms prior to the lighting of mosquito coils. This served as control and helped to determine the actual amount of particulates attributable to the burning of coils. Particulate matter emissions from the mosquito coils were monitored continuously in the indoor environment until the coil has completely burnt out. The measurements were logged at 1 min intervals at a flow rate of 2 L/min and average concentrations were estimated for approximately 8 h of monitoring. Logged data were downloaded onto a computer using DustcommSeries7 software.

### Risk characterization

The information generated from the mosquito coil emission experiments and data reported by Hogarh et al. [3] were applied in a Monte Carlo simulation using @RISK (“at risk”) software version 7.5 (Palisade Corporation, NY, USA) to generate additional scenarios. The @RISK shows possible outcomes for different scenarios and the likelihood of occurrence. By sampling different possible inputs, @RISK calculates thousands of possible future outcomes. The simulations were run with 100,000 iterations and the resultant data applied to evaluate the risks, in terms of the Hazard Index (HI) and Hazard Quotient (HQ) that may be associated with inhalation of emissions from a lit mosquito coil in the indoor environment.

1 $${\text{HI}}\; =\;\frac{{{\text{Concentration}}\;{\text{intake}}\; ( {\text{CI)}}}}{{{\text{Reference}}\;{\text{dose}}\; ( {\text{RfD)}}}}$$

2 $${\text{CI}}\;{ = }\;\frac{{{\text{Conc}} .\; \times \;{\text{EF}}\; \times \;{\text{ED}}\; \times \;{\text{IR}}\; \times {\text{P}}\; \times \;{\text{CF}}}}{{{\text{BW}}\; \times \;{\text{AT}}}}$$

where conc is the concentration of pollutant, EF is the exposure frequency, ED is the exposure duration, IR is the intake rate, P is the permeability rate, CF is the conversion factor, BW is the body weight, AT is the average time (period over which exposure is averaged)

3 $${\text{HQ }} = {\text{ HI}}_{\text{CO}} + {\text{ HI}}_{\text{TVOC}} + {\text{ HI}}_{{{\text{SO}}_{2} }} + {\text{ HI}}_{{{\text{NO}}_{2} }} + {\text{ HI}}_{{{\text{PM}}_{2.5} }} + {\text{ HI}}_{{{\text{PM}}_{10} }}.$$

## Results and discussion

### Susceptibility of mosquitoes to lit coils

**Fig. 1:**
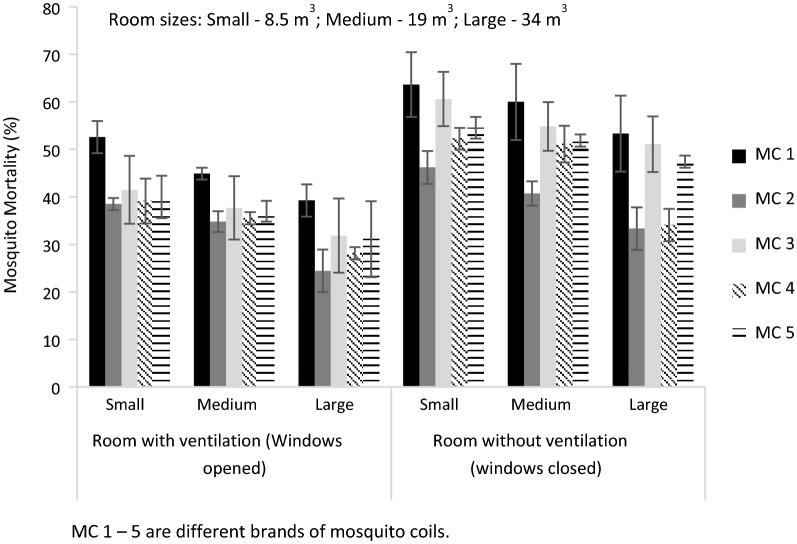
Mosquito mortality (% ± SD) induced by different mosquito coils under varying room volume and ventilation condition

Mosquito mortality resulting from the application of mosquito coils under the various experimental treatments ranged between 24 and 64% (Fig. 1). This does not signify adequate repellency effect. The recorded mortalities in this study were nevertheless relatively greater than those reported by Ogoma et al. [17], which averaged around 16%. According to WHO criteria for susceptibility test, 98–100% mortality suggests that susceptibility is adequate; 80–97% mortality means resistance is suspected with more investigations required; 0–79% mortality indicates resistance is confirmed [12]. Thus, the range of mortalities observed in this study has implications for potential resistance development of mosquitoes to insecticides. The mortalities were relatively greater when windows of experimental chambers were closed and there was poor ventilation (Fig. 1). Statistically, there was a significant difference between the mortalities of mosquitoes under ventilated and poorly ventilated conditions (p < 0.01). The mosquito coil MC1, which had the highest insecticidal effect (Fig. 1), has esbiothrin as the indicated active ingredient (Table 1). Statistically, there was no significant difference in mosquito mortality among the tested coils under ventilated conditions, but the difference was significant when rooms were poorly-ventilated (p < 0.05 in each case). The effectiveness of the mosquito coil in inducing mortality appeared to marginally decrease with an increase in room volume, i.e. from the 8.5 to 34 m^3^ rooms. Relatively increased mosquito mortalities ranged 69–86% were reported in experiments conducted with much smaller chamber volume of approximately 0.34 m^3^ [18, 19].

### Emissions from mosquito coils

#### Carbon monoxide (CO)

**Fig. 2:**
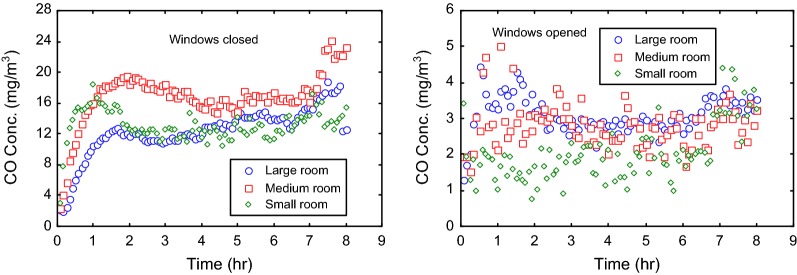
CO emission patterns associated with the burning of mosquito coils indoors

On the average, CO emissions from burning mosquito coils were 2.07, 2.82 and 3.08 mg/m^3^ in the small, medium and large rooms, respectively, when the windows of the experimental rooms were opened to allow natural ventilation (Table 4). When the experiment was repeated with windows closed, which is often the practice when people slept while burning coils at night, the concentrations of CO in the rooms expectedly increased. Under the closed room conditions, the average concentration of CO in the small, medium and large rooms were 13.18, 17.29 and 12.44 mg/m^3^, respectively (Table 4), representing increments by approximately a factor of 4–6 relative to the levels in ventilated rooms. Mean CO concentration of 6.5 ppm was previously reported following the application of mosquito coils in a closed indoor environment [11]. The time profiles of CO emissions showed that rooms with natural ventilation had quite an erratic pattern as a result of effect from air exchange (Fig. 2).

**Table 4:** Average concentrations (mg/m^3^) of indoor air pollutants from burning of mosquito coils

Mosquito coil	Ventilated condition^b^						Poorly ventilated condition^c^					
	CO	TVOC	NO_2_	SO_2_	PM_2.5_	PM_10_	CO	TVOC	NO_2_	SO_2_	PM_2.5_	PM_10_
	Room size: 8.5 m^3^						Room size: 8.5 m^3^					
MC 1	1.91	0.05	0.08	0.34	0.02	0.15	17.93	0.12	0.07	0.90	0.11	0.19
MC 2	1.95	0.05	0.07	0.16	0.07	0.04	7.56	0.13	0.07	0.66	0.16	0.15
MC 3	1.83	0.08	0.08	0.24	0.07	0.05	11.20	0.14	0.07	0.36	0.41	0.32
MC 4	2.51	0.04	0.07	0.29	0.08	0.18	8.12	0.16	0.07	0.43	0.63	0.67
MC 5	2.15	0.07	0.08	0.20	0.03	0.03	21.09	0.15	0.07	0.86	0.09	0.09
Average	2.07	0.06	0.08	0.25	0.06	0.09	13.18	0.14	0.07	0.64	0.28	0.28
Control	BDL	BDL	0.07	BDL	0.02	0.03	BDL	0.05	0.06	0.16	0.07	0.05
	Room size: 19 m^3^						Room size: 19 m^3^					
MC 1	2.66	0.03^a^	0.08	0.37	0.04	0.11	18.75	0.12	0.07	0.99	0.17	0.24
MC 2	1.99	0.04	0.08	0.19	0.06	0.06	12.09	0.18	0.06	0.68	0.23	0.20
MC 3	5.04	0.03^a^	0.08	0.16	0.09	0.05	10.15	0.19	0.06	0.38	0.31	0.44
MC 4	1.30	0.04	0.08	0.26	0.13	0.24	12.85	0.25	0.06	0.51	1.02	1.20
MC 5	3.10	0.06	0.08	0.13	0.04	0.03	32.60	0.19	0.07	1.00	0.23	0.10
Average	2.82	0.04	0.08	0.22	0.07	0.10	17.29	0.19	0.06	0.71	0.39	0.44
Control	BDL	BDL	0.07	BDL	0.02	0.02	BDL	0.05	0.05	0.25	0.06	0.06
	Room size: 34 m^3^						Room size: 34 m^3^					
MC 1	2.33	0.03^a^	0.07	0.23	0.04	0.15	14.21	0.12	0.06	0.96	0.09	0.19
MC 2	2.61	0.06	0.08	0.15	0.04	0.04	7.73	0.12	0.07	0.64	0.13	0.17
MC 3	4.91	0.06	0.07	0.26	0.06	0.10	9.86	0.12	0.07	0.44	0.23	0.33
MC 4	3.13	0.06	0.08	0.23	0.08	0.15	8.25	0.12	0.07	0.38	0.58	0.63
MC 5	2.42	0.05	0.08	0.25	0.06	0.02	22.15	0.11	0.08	0.86	0.15	0.14
Average	3.08	0.05	0.08	0.22	0.06	0.09	12.44	0.12	0.07	0.66	0.24	0.29
Control	BDL	BDL	0.07	BDL	0.03	0.02	BDL	0.06	0.06	0.15	0.05	0.04

*BDL* below detection limit

^a^Detection limit of TVOC gas sensor

^b^Windows opened; ^c^Windows closed

**Fig. 3:**
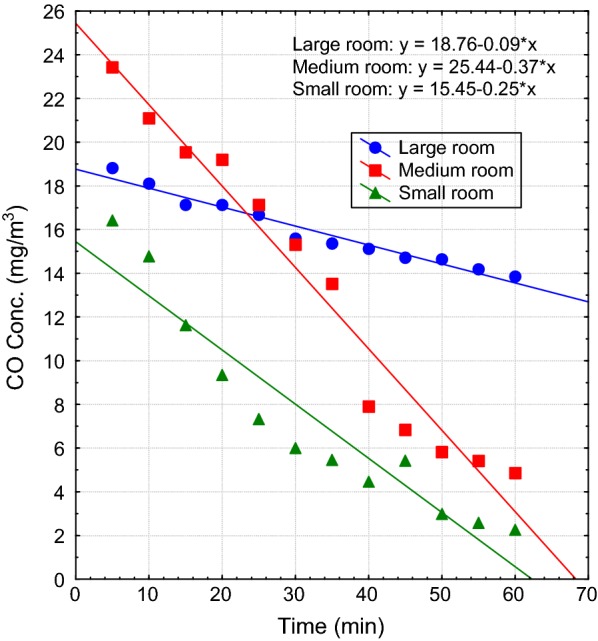
Rate of removal of CO from poorly aerated indoor environment after the use of mosquito coil

The profiles were more stable with less interference from outside air when windows were closed and air exchange was largely blocked. This led to accumulation of indoor levels of CO exceeding the WHO guideline of 10 mg/m^3^ for an 8-h average exposure [20]. The surge in CO concentration when windows were closed was statistically significant for the various rooms (approximately p = 0.01 in each instance). Considering that there are instances when mosquito coil could burn out completely while the user was still asleep and windows remained closed, post-burning period was monitored for 1 h to evaluate the decline of CO to safe levels. The average rates of decline of CO from the various rooms after the mosquito coils had finished burning were 0.25, 0.37 and 0.09 mg/m^3^ per min for the small, medium and large room, respectively (Fig. 3). Thus, the decline was slowest in the large room. At those rates, it took approximately 20 and 40 min for CO levels to reduce to about 10 mg/m^3^ in the small and medium rooms, respectively, but over an hour to reach such safe levels in the large room. Under circumstances of lack of ventilation, indoor air pollutant removal is likely to be influenced by diffusion mechanisms [21]. It is presumed that it took a relatively longer period for CO to diffuse out of the large room, hence, its slow rate of removal from this room. This has implications for risk of exposure to the pollutant during post-burning periods. It is notable that CO emissions were much elevated in some specific coil brands than others. For instance, MC1 and MC5 generated greater emissions relative to other coils under the closed indoor conditions (Table 4). This may be due to differences in the filler used in producing different mosquito coils. The present findings suggest that burning one strand of mosquito coil in a closed indoor environment yielded CO emissions of an order of magnitude quite comparable to the rate emanated from environmental tobacco smoke of 14–23 mg CO/cigarette [22].

#### Total volatile organic compounds (TVOCs)

**Fig. 4:**
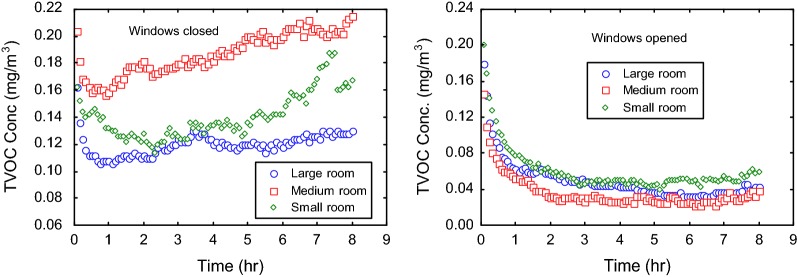
TVOC emission patterns associated with the burning of mosquito coils indoors

The sum of individual volatile organic compounds (VOCs) in a sample of air is usually referred to as TVOCs. TVOC concentrations reduced as the burning of mosquito coils proceeded in the indoor environment when the rooms were opened to natural ventilation; from initial concentrations of 0.15–0.20 mg/m^3^ to concentrations in the range of 0.025–0.075 mg/m^3^ within 2 h of burning (Fig. 4). Air exchange clearly removed VOCs from the rooms. The TVOC levels were relatively greater in the room without ventilation, with levels dropping marginally initially, but increasing gradually with time. The average concentrations of TVOCs in the ventilated rooms were 0.06, 0.04 and 0.05 mg/m^3^, and in non-ventilated rooms were 0.14, 0.19 and 0.12 mg/m^3^, for the small, medium and large rooms, respectively (Table 4). Thus, opening up windows for natural ventilation during the burning of the coils reduced TVOCs in the rooms by one order of magnitude compared to non-ventilated rooms. The variation of TVOCs with room volume under the non-ventilation conditions was statistically significant (p = 0.007). A suite of VOCs has been reported in mosquito coil smoke, with methylene chloride, benzene and toluene among the predominant compounds [6, 7]. Although the present study did not analyse individual VOCs, it was observed that the aggregate of VOCs in mosquito coil smoke in terms of TVOCs did not exceed 0.2 mg/m^3^, which meets the guideline of 0.3 mg/m^3^ as 8-h average stipulated in the UK for instance [23].

#### Sulfur dioxide (SO_2_)

**Fig. 5:**
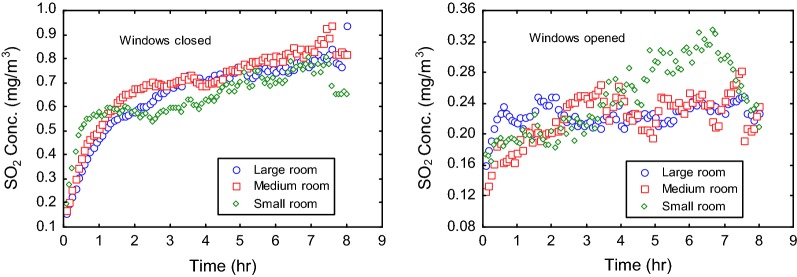
SO_2_ emission patterns associated with the burning of mosquito coils indoors

In developing countries, SO_2_ in the indoor environment has traditionally been linked to the use of poor fuels such as charcoal, firewood and kerosene [24–27]. The present study revealed that burning of mosquito coils, especially when used on regular basis, may add up to source factors of exposure to SO_2_ in the indoor environment in developing countries. The concentration of SO_2_ in ventilated rooms was averagely 0.25, 0.22 and 0.22 mg/m^3^ for the small, medium and large rooms, respectively (Table 4). The levels were expectedly greater under closed room conditions at respective concentrations of 0.64, 0.71 and 0.66 mg/m^3^. Thus, allowing ventilation while burning mosquito coils reduced the indoor levels of SO_2_ by approximately a factor of 3. Generally, the time profile of SO_2_ emissions showed increases in pollutant concentration with time during the burning of the coil. This increasing trend was marginal for ventilated rooms and relatively pronounced under closed conditions (Fig. 5). Some brands of mosquito coil, particularly MC1, consistently generated relatively elevated levels of SO_2_ (Table 4). Emissions of SO_2_ from the coils are indicative of sulfur containing materials as part of the base materials of the coil products. It is presumed that mosquito coil products with relatively greater content of sulfur emitted comparatively greater amount of SO_2_. Nevertheless, the emissions were within short-term exposure limit for SO_2_ set at 0.05 mg/m^3^ [28].

#### Nitrogen dioxide (NO_2_)

**Fig. 6:**
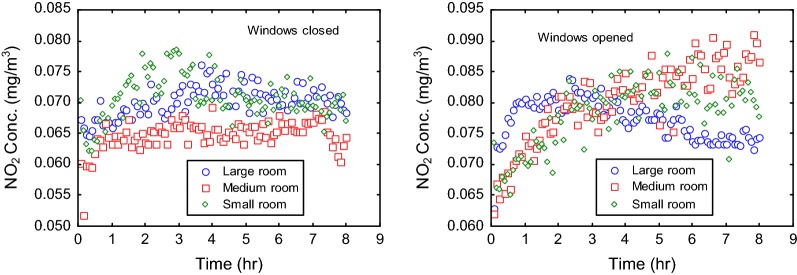
NO_2_ emission patterns associated with the burning of mosquito coils indoors

The time profiles of NO_2_ emissions from the burning coils indicated slightly elevated range of NO_2_ concentrations in ventilated rooms compared to rooms with closed windows (Fig. 6). The 8-h average concentration of NO_2_ was mostly 0.08 mg/m^3^ for the various experimental rooms under ventilation, and between 0.06 and 0.07 when air exchange was largely blocked. The controls (that is, indoor levels of NO_2_ without burning of mosquito coils) similarly showed relatively increased concentration of NO_2_ when windows were opened for ventilation (Table 4). This result, thus, suggests that the levels of NO_2_ in the indoor measurement were largely contributed from the external air rather than the burning mosquito coil. This is consistent with other findings in which NO_2_ was largely undetected in mosquito coil smoke [7]. It is indicative of absence of nitrogenous material in the base materials of most mosquito coil products.

#### Particulate matter (PM)

**Fig. 7:**
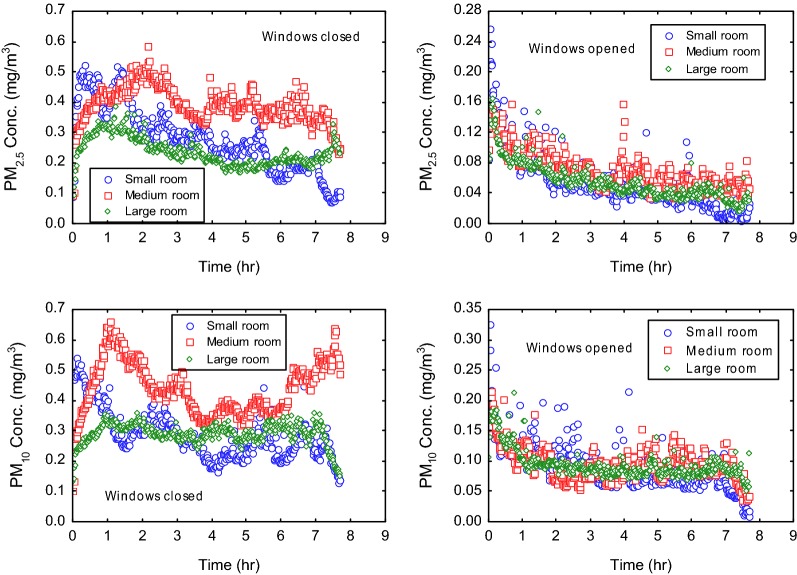
PM_2.5_ and PM_10_ emission patterns associated with the burning of mosquito coils indoors

The concentration of particulate matter in indoor air resulting from the burning of mosquito coils is shown in Fig. 7. The burning of the mosquito coils under a poor ventilation situation when windows were closed, yielded as 8-h averages, PM_2.5_ concentrations of 0.28, 0.39 and 0.24 mg/m^3^ for room space of 8.5, 19 and 34 m^3^, respectively (Table 4). These were generally above PM_2.5_ 8-h threshold emissions of 0.04 mg/m^3^ [28]. The variation in concentration of PM_2.5_ with room size was not statistically significant (p = 0.65). When the experiment was repeated under natural ventilation with windows opened, the concentration of PM_2.5_ reduced to 0.06, 0.03 and 0.06 mg/m^3^, for the respective rooms. The reduction in PM_2.5_ levels with ventilation was statistically significant (p = 0.05 in each case). Expectedly, PM_10_ fractions of the particulate emissions were also relatively greater under conditions of poor ventilation (Table 4). The 8-h averages of PM_10_ for the small, medium and large rooms were 0.28, 0.44 and 0.29, respectively, above the acceptable threshold of 0.05 mg/m^3^ in Germany [23]. The variation of PM_10_ levels with room size was not statistically significant (p = 0.69). The reduction in PM_10_ levels while burning mosquito coils with windows opened for ventilation was statistically significant for only the large room (p = 0.04). This suggests reduced removal of PM_10_ from the smaller rooms, even when the mosquito coils were applied with windows opened for ventilation.

### Risk–benefit analysis of mosquito coil application

The task was to model the benefits and risks of application of mosquito coils as a malaria control method. The potential benefit of applying mosquito coil hinges on its ability to repel/kill mosquitoes. As realized from this study, the mosquito coil induced not more than 70% mosquito mortality. This does not sufficiently protect against potential mosquito bites. At the same time, application of the mosquito coil was characterized by emissions of CO, TVOC, NO_2_, SO_2_, PM_2.5_ and PM_10_ (Figs. 2, 4, 5, 6 and 7), which could present respiratory and other health related risks. Applying Eqs. 1 and 2, the HI for the various pollutants were estimated for 100,000 iterations with summarized data presented in Tables 5 and 6. In applying Eqs. 1 and 2, the concentrations of pollutants were derived from Table 4; EF was derived from the average number of coils used per week [3]; ED was 6–8 h depicting the approximate duration of sleep while using mosquito coil; IR was assumed as 15 m^3^/d [29]; the permeability rate was assumed to be 1, which reflects a worst case scenario; BW was assumed to be 65–70 kg. The risk was averaged over 60 years, considering that the approximate life expectancy of an individual in Ghana is about 60 years [30]. The HI for the various pollutants were generally low (less than 1), which suggests that the individual pollutants emitted from the mosquito coil may not constitute adverse environmental health risk over a lifetime of opportunistic exposures. The mean HI of each pollutant was relatively greater under conditions of poor ventilation (Tables 5 and 6). Among the pollutants, the highest risk was associated with CO. Approximately 3 persons in one billion (under the ventilation scenario) and 2 persons in 100 million in a poorly ventilated indoor environment may suffer CO mobidity from the use of mosquito coil in Ghana. Applying Eq. 3, the HQ, which is an aggregate of the risks from the individual pollutants, was estimated as 1.4 × 10^−8^ and 6.6 × 10^−8^ for ventilated and poorly ventilated indoor environments, respectively. A risk of 1.4 × 10^−8^ represents 1.4 morbidity per 100,000,000 persons; but because we cannot have a fraction of a person, it was approximated to 2 per 100,000,000 persons. By similar explanation, 6.6 × 10^−8^ represents a risk of 7 morbidity per 100,000,000 persons. Thus, when all the pollutants were considered, approximately 2 persons in 100 million (under a ventilation scenario) and 7 persons in 100 million (under poor ventilation scenario) may suffer from the use of mosquito coil in Ghana. There is presently no HQ safety reference value for mosquito coil emissions. However, from risk assessment perspective, a risk of 1 × 10^−6^ (i.e. 1 per 1,000,000 persons) is sufficiently low and acceptable.

**Table 5:** Hazard index (HI) of potential exposure to indoor air pollutants emitted from mosquito coils in relatively ventilated indoor environment

Pollutant	Min	Max	Mean	Std dev	5th percentile	95th percentile
CO	5.0 × 10^−14^	2.9 × 10^−8^	2.7 × 10^−9^	2.4 × 10^−9^	1.7 × 10^−10^	7.4 × 10^−9^
TVOC	4.1 × 10^−16^	3.5 × 10^−10^	5.0 × 10^−11^	4.3 × 10^−11^	3.3 × 10^−12^	1.4 × 10^−10^
NO_2_	8.6 × 10^−16^	3.5 × 10^−10^	7.7 × 10^−11^	6.0 × 10^−11^	5.4 × 10^−12^	1.9 × 10^−10^
SO_2_	2.7 × 10^−15^	2.4 × 10^−9^	2.3 × 10^−10^	2.1 × 10^−10^	1.5 × 10^−11^	6.3 × 10^−10^
PM_2.5_	0	9.1 × 10^−10^	6.2 × 10^−11^	6.0 × 10^−11^	3.5 × 10^−12^	1.8 × 10^−10^
PM_10_	1.7 × 10^−15^	2.0 × 10^−9^	9.0 × 10^−11^	1.2 × 10^−10^	3.5 × 10^−12^	3.1 × 10^−10^

**Table 6:** Hazard index (HI) of potential exposure to indoor air pollutants emitted from mosquito coils in indoor environment lacking adequate ventilation

Pollutant	Min	Max	Mean	Std dev	5th percentile	95th percentile
CO	1.3 × 10^−13^	3.6 × 10^−6^	1.7 × 10^−8^	3.7 × 10^−8^	8.3 × 10^−10^	4.8 × 10^−8^
TVOC	1.4 × 10^−15^	4.9 × 10^−9^	1.5 × 10^−10^	1.4 × 10^−10^	10.0 × 10^−12^	4.0 × 10^−10^
NO_2_	7.8 × 10^−16^	3.8 × 10^−10^	7.1 × 10^−11^	5.6 × 10^−11^	4.9 × 10^−12^	1.8 × 10^−10^
SO_2_	1.0 × 10^−14^	4.7 × 10^−9^	6.9 × 10^−10^	6.0 × 10^−10^	4.3 × 10^−11^	1.9 × 10^−9^
PM_2.5_	2.4 × 10^−15^	8.5 × 10^−6^	8.5 × 10^−10^	3.1 × 10^−8^	1.3 × 10^−11^	1.6 × 10^−9^
PM_10_	1.4 × 10^−15^	7.6 × 10^−9^	3.3 × 10^−10^	1.4 × 10^−10^	1.4 × 10^−11^	1.1 × 10^−9^

## Conclusion

The susceptibility of mosquitoes to mosquito coils, ranged between 24 and 64% mortality; representing inadequate spatial repellency effect. The range of mortalities observed in this study has implications for the mosquitoes’ potential development of resistance to insecticides. Efficacy of the mosquito coil in inducing mortality appeared to marginally decrease with an increase in room volume. The effect, however, relatively increased in poorly ventilated rooms. Several pollutants including CO, TVOCs, SO_2_, NO_2_ and particulate matter (PM_2.5_ and PM_10_) were detected in the indoor air following application of mosquito coil. But the aggregate risk from these pollutants, expressed in terms of Hazard Quotient, was relatively low and estimated at 1.4 × 10^−8^ and 6.6 × 10^−8^ for the ventilated and poorly ventilated indoor environments, respectively. Although the risk of morbidity from the use of the mosquito coil per the emissions generated was characterized to be low, coil use produced limited protection against mosquito bites. It may therefore have a reduced benefit as malaria prevention and control strategy; hence, its application in malaria endemic regions should be re-assessed.
